# Impact of pullback speed on evaluation of lipid core plaque using near-infrared spectroscopy-intravascular ultrasound

**DOI:** 10.1007/s12928-025-01124-7

**Published:** 2025-04-03

**Authors:** Tetsuharu Kasahara, Hideki Kitahara, Kenta Takou, Kazuya Tateishi, Yuichi Saito, Ken Kato, Takashi Iimori, Yoshio Kobayashi

**Affiliations:** 1 Department of Radiology, Chiba University Hospital, Chiba, Japan; 2 Department of Cardiovascular Medicine, Chiba University Graduate School of Medicine, 1-8-1 Inohana, Chuo-ku, Chiba, Chiba 260-8677 Japan

**Keywords:** Near-infrared spectroscopy, Intravascular ultrasound, Lipid core burden index, Pull-back speed

## Abstract

Near-infrared spectroscopy-intravascular ultrasound (NIRS-IVUS) can assess the amount of lipid core plaque, and the latest generation catheter allows for imaging at several different pullback speeds. The purpose of this study was to investigate the impact of pullback speed on evaluation of lipid core plaque using the latest generation NIRS-IVUS catheter. Patients with coronary artery disease who underwent coronary angiography or percutaneous coronary intervention with NIRS-IVUS examination at 2 different pullback speeds (0.5 and 2.0 mm/sec) for the same vessel were enrolled. The values of lipid core burden index (LCBI) and maxLCBI4mm were compared between the images recorded with pullback speeds of 0.5 and 2.0 mm/sec, and match or mismatch of the location where maxLCBI4mm was detected was investigated. In a total of 286 NIRS-IVUS image sets, the location of maxLCBI4mm measured at 0.5 and 2.0 mm/sec was mismatched in 81 (28.3%) image sets. In comparison by vessel type, 20.8% were mismatched in LAD, 31.1% in LCx, and 37.8% in RCA (*p* = 0.023). In 205 image sets with matched maxLCBI4mm location, the maxLCBI4mm value at 2.0 mm/sec was significantly greater compared with that at 0.5 mm/sec (348.0 vs. 302.0 in median, *p* < 0.001). In a substantial proportion of patients, the location of maxLCBI4mm values measured at 0.5 and 2.0 mm/sec was mismatched. In the image sets with matched maxLCBI4mm location, the maxLCBI4mm value at 2.0 mm/sec was significantly greater compared with that at 0.5 mm/sec. These results should be noted when applying the cut-off values of maxLCBI4mm recorded at 2.0 mm/sec.

**Graphical abstract:**

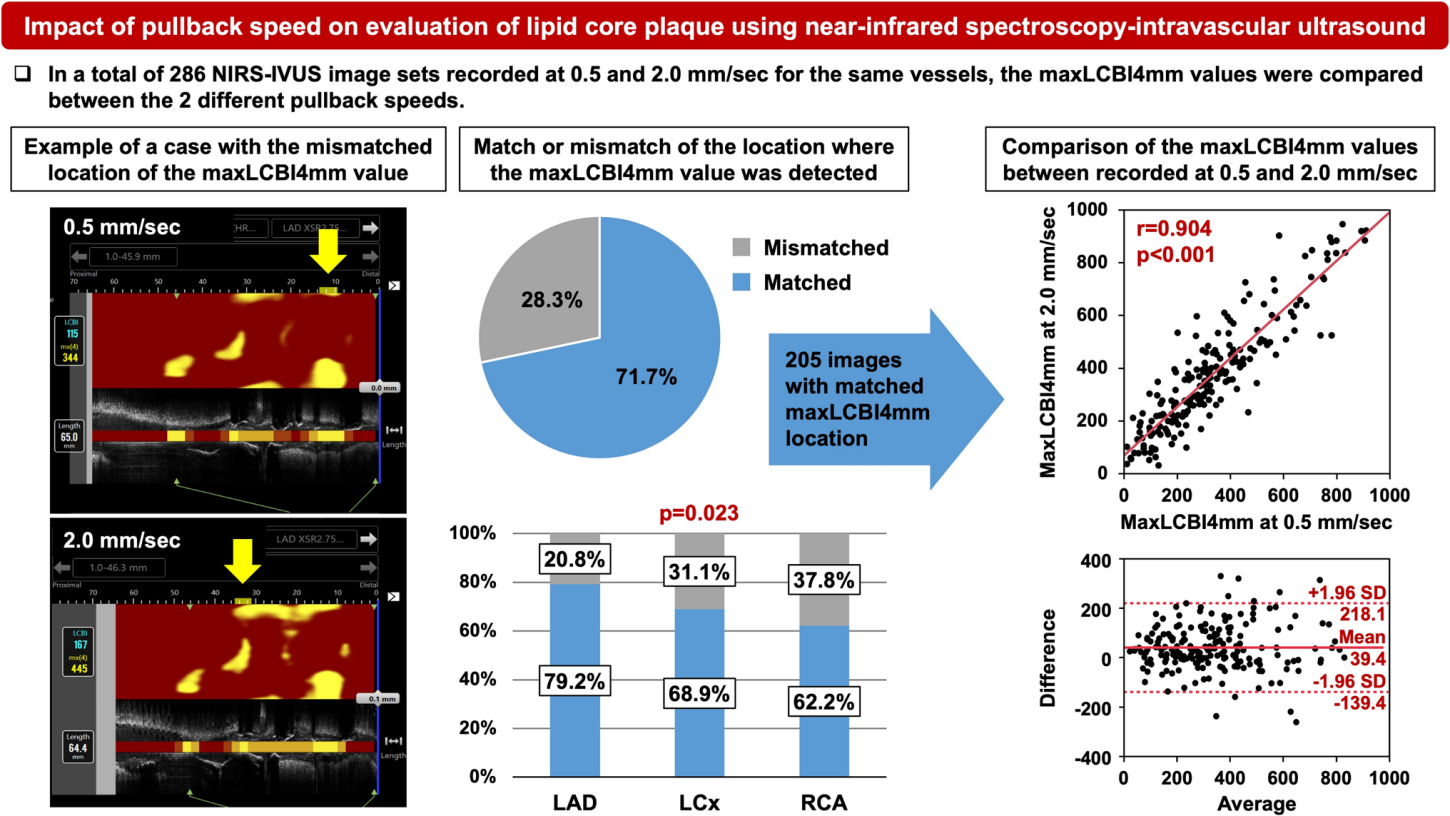

## Introduction

Intravascular imaging, such as intravascular ultrasound (IVUS) and optical coherence tomography, has become important modalities for successful percutaneous coronary intervention (PCI) procedures in recent years [1–8]. Near-infrared spectroscopy (NIRS)-IVUS, consisting of an ultrasound transducer and a scanning near-infrared laser, can detect the lipid content of atherosclerotic plaque and quantitatively assess the amount of lipid core plaque. The degree of lipid core plaque is described as lipid core burden index (LCBI), and especially, the highest LCBI contained in the 4-mm segment (maxLCBI4mm) has been reported to have a predictive value for future cardiovascular events [9–12] as well as periprocedural myocardial infarction in patient with coronary artery disease [13–15]. A previous study to evaluate the accuracy of lipid component quantification by NIRS-IVUS was first conducted using coronary autopsy specimens [16], and it was further validated in living patients [17]. In addition, intra- and inter-catheter reproducibility of measurement using NIRS-IVUS catheters has appropriately been confirmed in the previous independent studies [18, 19]. Since the pullback speed of NIRS-IVUS catheter in the early generation was only 0.5 mm/sec, most of those basic data were all acquired at 0.5 mm/sec. Therefore, the pullback speed of 0.5 mm/sec has been used in major clinical trials evaluating the clinical usefulness of NIRS-IVUS [9–11, 20]. On the other hand, the latest generation catheter, the Makoto system, allows for 3 different pullback speeds: 0.5, 1.0, and 2.0 mm/sec [21]. In routine clinical practice, it is often used at a faster pullback speed to save time during the PCI procedure. However, the reproducibility of different pullback speeds has not been validated in the latest generation system in which the analysis algorithm for LCBI originally established using data recorded at 0.5 mm/sec is applied to 2.0 mm/sec. Thus, the purpose of this study was to investigate the impact of pullback speed on LCBI values evaluated using the latest generation NIRS-IVUS catheter.

## Methods

### Study population

Patients with coronary artery disease who underwent coronary angiography (CAG) or PCI with NIRS-IVUS examination at 2 different pullback speeds (0.5 and 2.0 mm/sec) for the same vessel at Chiba University Hospital between October 2018 and October 2021 were prospectively investigated. Of 344 IVUS pullback image sets in 116 patients, images in which only left main coronary artery was observed (*n* = 34), those with the maxLCBI4mm value of 0 measured at either pullback speed (*n* = 23), and that with mismatch of the locations recorded at 2 pullback speeds (*n* = 1), were excluded. Consequently, 286 NIRS-IVUS image sets in 114 patients were included in this analysis. All patients provided written informed consent for the examination, and the ethical committee of Chiba University Graduate School of Medicine approved the present study (unique identifier: 2646).

### NIRS-IVUS imaging

The NIRS-IVUS system with a 3.2 Fr rapid exchange catheter, auto-pullback controller, and console (Makoto^™^ Intravascular Imaging System, InfraReDx, Inc., Bedford, MA, USA) was used. NIRS-IVUS images were acquired under various circumstances whenever possible during the PCI and CAG procedure. In addition to NIRS-IVUS imaging of the target vessel at PCI, observation of non-target vessels was conducted as much as possible, and vessels with stenotic lesions at CAG was also observed at the operator’s discretion. After intra-coronary administration of isosorbide dinitrate 1 mg, NIRS-IVUS imaging was performed as distally as possible in the entire target vessel. NIRS-IVUS images were recorded with motorized transducer pullback speed of 0.5 mm/sec, followed by recording with pullback speed of 2.0 mm/sec at the same location without moving the catheter. NIRS data were analyzed with the console of NIRS-IVUS. The values of LCBI, which can be calculated in any observed segment of interest, and maxLCBI4mm, which is the 4-mm segment with the highest LCBI in the segment of interest, were collected. The agreement of the analyzed segments of interest was carefully confirmed by correlating anatomical features (such as side branch, calcification, vein running around the artery, etc.) between the images recorded at 2 different pullback speeds. The values of LCBI and maxLCBI4mm were compared between the images recorded with pullback speeds of 0.5 and 2.0 mm/sec, and match or mismatch of the location where maxLCBI4mm was detected was also investigated (Table 1).

**Table 1:** Comparison between the matched and mismatched maxLCBI4-location groups

	Matched (*n* = 205)	Mismatched (*n* = 81)	*p* value
Vessel			
LAD	103 (50.2)	27 (33.3)	0.023
LCx	51 (24.9)	23 (28.4)	
RCA	51 (24.9)	31 (38.3)	
Situation			
Vessels observed at CAG	37 (18.1)	8 (9.9)	0.015
Non-target vessels at PCI	54 (26.3)	33 (40.7)	
Target vessels before stenting at PCI	66 (32.2)	16 (19.8)	
Target vessels after stenting at PCI	48 (23.4)	24 (29.6)	
Analyzed vessel length, mm			
0.5 mm/sec	59.4 (43.5–77.9)	63.4 (45.9–79.3)	0.623
2.0 mm/sec	59.0 (43.7–77.8)	62.0 (41.6–80.4)	0.785
HR, bpm			
0.5 mm/sec	68.0 (62.0–75.0)	69.0 (57.0–79.0)	0.350
2.0 mm/sec	69.0 (62.0–76.0)	67.0 (56.5–76.0)	0.105

Data are given as the median (interquartile range) or *n* (%)

*CAG* coronary angiography, *HR* heart rate, *LAD* left anterior descending artery, *LCBI* lipid core burden index, *LCx* left circumflex artery, *PCI* percutaneous coronary intervention, *RCA* right coronary artery

### Statistical analysis

Statistical analysis was performed using JMP^®^ 18.0 (SAS Institute, Cary, NC, USA). Categorical variables are presented as percentages and compared between the 2 groups using Pearson's chi-squared test. Continuous variables are presented as median (interquartile range). Comparisons of continuous variables between the 2 groups were conducted with Mann–Whitney U test, and comparisons among the 3 groups were done using Kruskal–Wallis test. Paired maxLCBI4mm values at 0.5 mm/sec and 2.0 mm/sec were compared with Wilcoxon signed-rank test. The relationship between maxLCBI4mm values at 0.5 mm/sec and 2.0 mm/sec were examined with Pearson’s correlation test. Bland–Altman method was employed to test the agreement between the maxLCBI4mm values at 0.5 mm/sec and 2.0 mm/sec. A *p* < 0.05 was considered statistically significant.

## Results

Overall, 114 patients with 286 vessels of NIRS-IVUS image sets were analyzed in this study. The average age was 68.6 ± 10.1 years, and 90 (78.9%) patients were male. NIRS-IVUS imaging was performed at CAG in 18 (15.8%) patients and at PCI procedure in 96 (84.2%) patients. In terms of the observed vessels in 286 NISR-IVUS image sets, 130 (45.5%) vessels were left anterior descending artery (LAD), 74 (25.9%) were left circumflex artery (LCx), and 82 (28.7%) were right coronary artery (RCA). About the situation in which NIRS-IVUS imaging was performed, the vessels observed at CAG were in 45 (15.7%) image sets, the non-target vessels at PCI were in 87 (30.4%) image sets, the target vessels before stenting at PCI were recorded in 82 (28.7%) image sets, and the target vessels after stenting at PCI were recorded in 72 (25.2%) image sets. The median values of analyzed vessel length were 61.4 (44.1–77.9) mm in the images recorded at 0.5 mm/sec and 60.8 (43.4–78.4) mm at 2.0 mm/sec (*p* = 0.604). The median values of heart rate (HR) during the recording were 69.0 (60.0–76.0) bpm at 0.5 mm/sec and 69.0 (60.0–76.0) bpm at 2.0 mm/sec (*p* = 0.745).

**Fig. 1:**
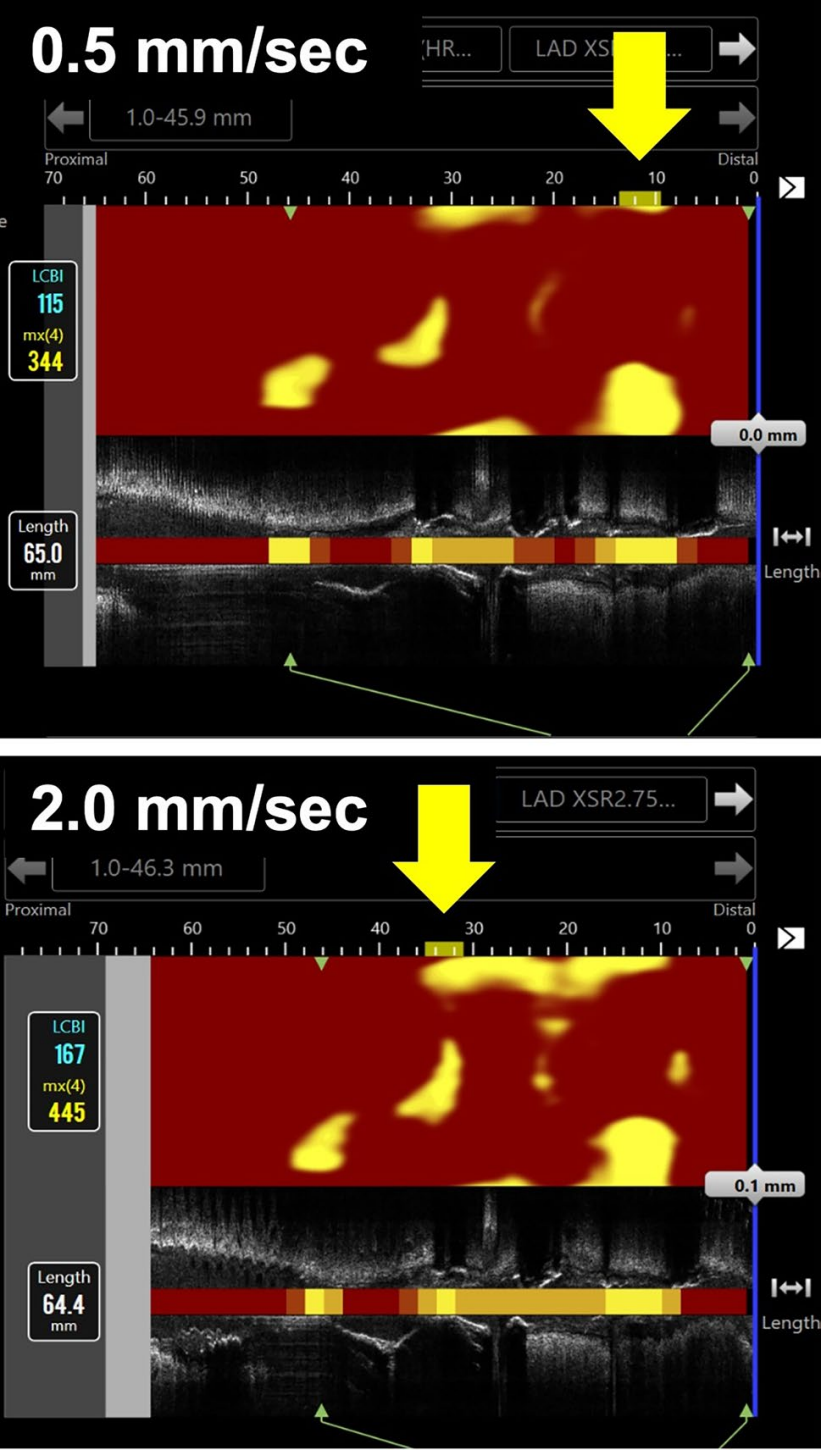
Representative images with the mismatched maxLCBI4mm location. Yellow arrows indicate the location of the maxLCBI4mm values. The maxLCBI4mm values were 344 and 445 in the images recorded at 0.5 mm/sec and at 2.0 mm/sec, respectively. *LCBI* lipid core burden index

The location of maxLCBI4mm was matched in 205 (71.7%) image sets, while it was mismatched in the remaining 81 (28.3%) image sets (Fig. 1). Lesion and procedural characteristics were compared between the maxLCBI4mm-location matched and mismatched groups (Table 1). The percentage of vessel type was significantly different between the matched and mismatched groups (*p* = 0.023), and the percentage of RCA was significantly higher in the mismatched group. In comparison by vessel type, 20.8% were mismatched in LAD, 31.1% in LCx, and 37.8% in RCA (*p* = 0.023). The type of imaging situation was significantly different between the 2 groups (*p* = 0.015), and the proportion of non-target vessels at PCI was higher in the mismatched group. The analyzed vessel length and heart rate were comparable between the 2 groups.

In 205 image sets with matched maxLCBI4mm location, maxLCBI4mm values were compared between the images at 0.5 mm/sec and 2.0 mm/sec (Table 2). Overall, the maxLCBI4mm value at 2.0 mm/sec was significantly greater compared with that at 0.5 mm/sec (*p* < 0.001). In each coronary artery, the maxLCBI4mm value at 2.0 mm/sec was similarly greater in LAD (*p* < 0.001), LCX (*p* = 0.015), and RCA (*p* < 0.001) compared with that at 0.5 mm/sec. In terms of the situation in which the maxLCBI4mm value was measured, the maxLCBI4mm value at 2.0 mm/sec was significantly higher in vessels observed at CAG (*p* < 0.001), in the non-target vessels at PCI (*p* = 0.009), and in the target vessels before stenting (*p* < 0.001) compared with that at 0.5 mm/sec, although it was comparable between the 2 pullback speeds in the target vessels after stenting (*p* = 0.211).

**Table 2:** MaxLCBI4mm values recorded at 0.5 mm/sec and 2.0 mm/sec

	0.5 mm/sec	2.0 mm/sec	*p* value
Overall (*n* = 205)	302.0 (187.0–438.0)	348.0 (219.0–482.5)	< 0.001
Artery			
LAD (*n* = 103)	310.0 (205.0–473.0)	366.0 (260.0–506.0)	< 0.001
LCX (*n* = 51)	289.0 (163.0–368.0)	292.0 (198.0–432.0)	0.015
RCA (*n* = 51)	272.0 (139.0–412.0)	348.0 (160.0–495.0)	< 0.001
Situation			
Vessels observed at CAG (*n* = 37)	279.0 (174.5–386.5)	319.0 (217.0–469.5)	< 0.001
Non-target vessels at PCI (*n* = 54)	286.0 (173.5–386.8)	306.0 (222.3–392.5)	0.009
Target vessels before stenting (*n* = 66)	400.5 (256.5–669.5)	475.5 (340.5–659.5)	< 0.001
Target vessels after stenting (*n* = 48)	232.5 (165.5–322.0)	245.0 (182.8–361.3)	0.211

Data are given as the median (interquartile range)

*CAG* coronary angiography, *LAD* left anterior descending artery, *LCBI* lipid core burden index, *LCx* left circumflex artery, *PCI* percutaneous coronary intervention, *RCA* right coronary artery

**Fig. 2:**
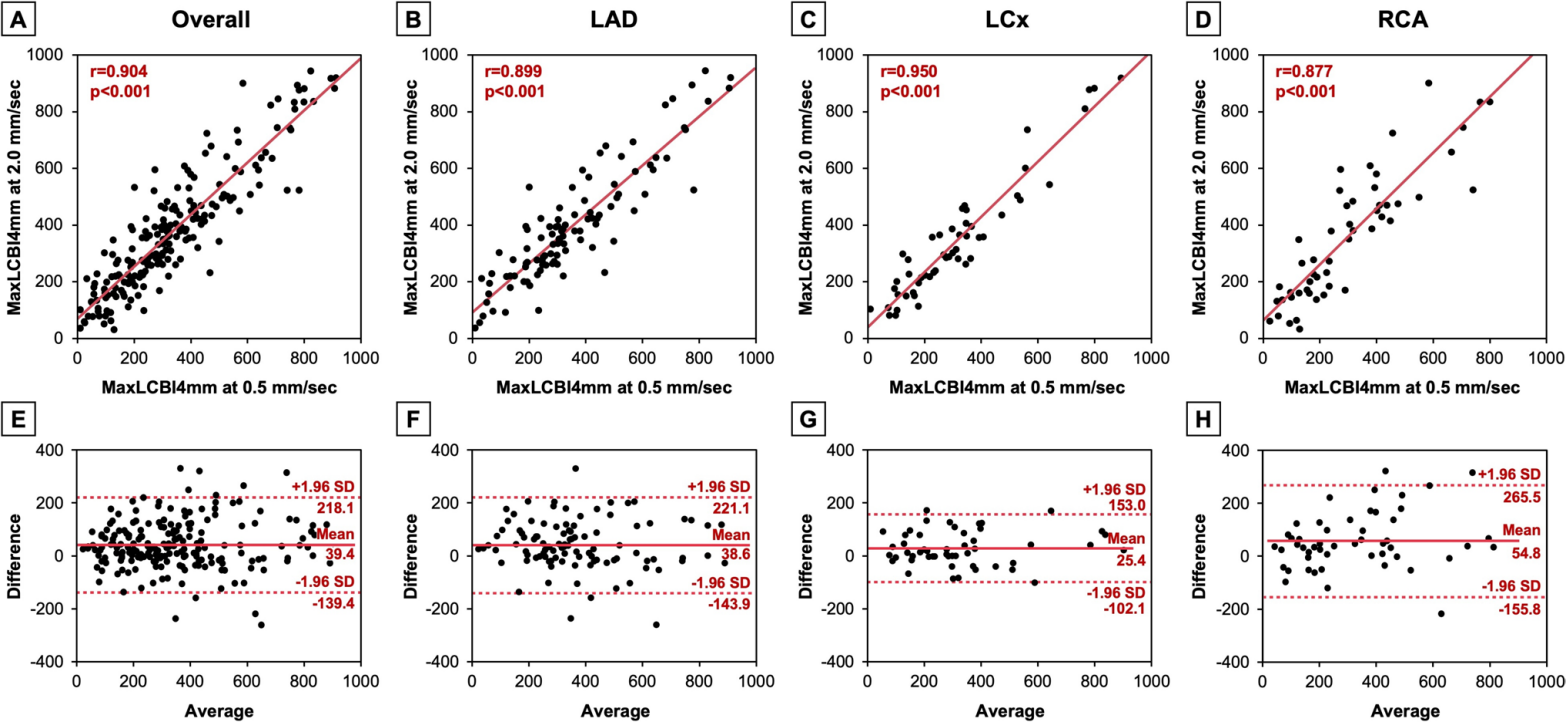
Comparison of maxLCBI4mm values between recorded at 0.5 and 2.0 mm/sec (overall and vessel type). The correlations and Bland–Altman analyses between maxLCBI4mm values recorded at 0.5 and 2.0 mm/sec in all images and in each vessel: LAD, LCx, and RCA. *LAD* left anterior descending artery, *LCBI* lipid core burden index; *LCx* left circumflex artery, *RCA* right coronary artery

**Fig. 3:**
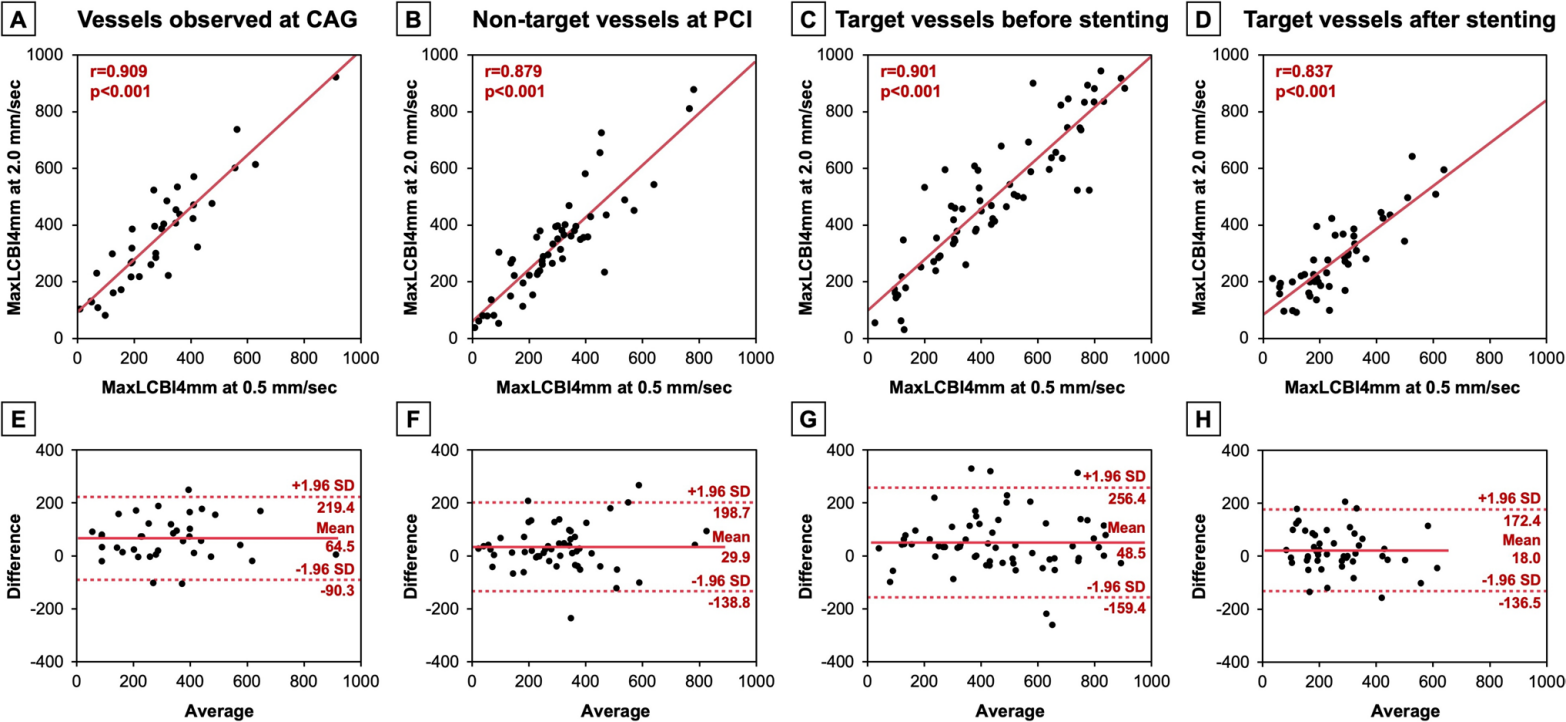
Comparison of maxLCBI4mm values between recorded at 0.5 and 2.0 mm/sec (imaging situation). The correlations and Bland–Altman analyses between maxLCBI4mm values recorded at 0.5 and 2.0 mm/sec in each imaging situation. *LCBI* lipid core burden index

The correlations and Bland–Altman analyses between maxLCBI4mm values recorded at 0.5 mm/sec and 2.0 mm/sec are presented in Fig. 2. Overall, there was a good correlation in maxLCBI4mm value recorded at 0.5 mm/sec and 2.0 mm/sec (*r* = 0.904). Correlation coefficients were slightly different among LAD (*r* = 0.899), LCx (*r* = 0.950), and RCA (*r* = 0.877). Bland–Altman analyses showed that limit of agreement was narrower in LCx compared with LAD and RCA. Similar analyses were conducted in each imaging situation as shown in Fig. 3. Good correlations were observed in vessels observed at CAG (*r* = 0.909), in the non-target vessels at PCI (*r* = 0.879), in the target vessels before stenting (*r* = 0.901), and in the target vessels after stenting (*r* = 0.837). Bland–Altman analyses demonstrated that limit of agreement was relatively wider in the target vessels before stenting compared with other 3 groups.

## Discussion

The main findings of the present study are as follows: (1) In a total of 286 NIRS-IVUS image sets, the location of maxLCBI4mm values measured at 0.5 mm/sec and 2.0 mm/sec was mismatched in 81 (28.3%) image sets; (2) Vessel type and imaging situation were associated with mismatch of the maxLCBI4mm location; (3) In 205 image sets with matched maxLCBI4mm location, the maxLCBI4mm value at 2.0 mm/sec was significantly greater compared with that at 0.5 mm/sec, irrespective of vessel type and imaging situation, except for the target vessels after stenting; and (4) The correlations and the limit of agreement by Bland–Altman analyses differed to some extent depending on vessel type and imaging situation.

Many previous studies have reported the usefulness of NIRS-IVUS, such as evaluation of culprit lesions in patients with acute coronary syndrome [22–25], estimation of periprocedural myocardial infarction at the PCI procedure [13–15], and prediction for future cardiovascular events [9–12]. Among such previous reports, several studies have proposed clinically useful cut-off values of maxLCBI4mm. In ACS patients, maxLCBI4mm > 400 has been reported as a signature of plaques causing ST-segment elevation myocardial infarction (STEMI) [23, 24]. Data from the COLOR registry have demonstrated that target lesions with a maxLCBI4mm ≥ 500 were associated with periprocedural myocardial infarction in as high as 50% of cases [13]. In previous reports on prognostic impact of NIRS-IVUS evaluation, detection of large LRP, maxLCBI4 mm ≥ 400, at non-stented sites in a target artery was associated with an increased risk of future major adverse cardiovascular and cerebrovascular events [26]. The LRP study has also revealed the maxLCBI4mm threshold value of 400 in non-culprit segments as a predictor of future patient-level MACE [11]. When applying the thresholds of maxLCBI4mm value as these previous studies have shown, the accuracy and reproducibility of the value should be essential. The accuracy of measurements using the NIRS-IVUS system was originally validated and established using data recorded at 0.5 mm/sec [16–19]. However, in routine clinical practice, it is often used at a faster pullback speed, 1.0 or 2.0 mm/sec, to save time during the catheterization procedure. Therefore, it would be important to verify whether maxLCBI4mm values recorded at 2.0 mm/sec can be treated as the same as those recorded at 0.5 mm/sec.

In the present study, the location of maxLCBI4mm measured at 0.5 mm/sec and 2.0 mm/sec was mismatched in 28.3% of image sets. In addition, the vessel type and imaging situation were associated with the occurrence of mismatch, whereas the analyzed vessel length and HR at examination were comparable between the matched and mismatched groups. In the mismatched group, the percentage of RCA was higher than in the matched group. It has been reported that RCA is more susceptible to motion artifacts during coronary artery imaging compared with LAD and LCX, because of its greater movement during the cardiac cycle, which can lead to increased image blurring and challenges in accurately assessing the vessel [27, 28]. Therefore, the relatively greater motion of RCA might have led to the results of this study. With respect to imaging situation, the percentage of non-target vessels at PCI was highest in the mismatched group. Although it is not clear why location mismatch of maxLCBI4mm often occurred in non-target vessels at PCI, these seems to be noteworthy results in clinical use of NIRS-IVUS imaging.

In the image sets with matched maxLCBI4mm location, the maxLCBI4mm value at 2.0 mm/sec was significantly greater than that at 0.5 mm/sec. Since the speed of the object being irradiated with near-infrared light may affect the reflection and scattering of the light, the faster pullback speed might result in the higher maxLCBI4mm values. However, the apparent reasons for this phenomenon are unknown, and thus, it is necessary to verify this issue in the future. In the RCA, the correlation coefficient between 0.5 mm/sec and 2.0 mm/sec was relatively lower and the limit of agreement by Bland–Altman analysis was wider compared with LAD and LCx. These results might be associated with the susceptibility of RCA to motion artifact, similarly to the mismatch of maxLCBI4mm location. Careful attention should be taken when measuring the maxLCBI4mm value especially in RCA. In terms of imaging situation, while there were some differences in correlation coefficients and the limit of agreement depending on the imaging situation, the values recorded at 2.0 mm/sec tended to be higher in all situations. These results for the measurements of maxLCBI4mm values should be noted when applying the cut-off values of maxLCBI4mm recorded at 2.0 mm/sec. When we find the borderline maxLCBI4mm value measured at 2.0 mm/sec, it may be better to measure again at 0.5 mm/sec at this time, because verification is still insufficient to adjust the cut-off value by considering the discrepancy between the values at 2.0 and 0.5 mm/sec.

### Limitations

There were several limitations in the present study. First, sample size was relatively small, especially in the groups divided by the vessel type and imaging situation. Second, this study did not examine the impact of plaque volume and plaque characteristics on the difference in the maxLCBI4mm value between 0.5 and 2.0 mm/sec. Third, the maxLCBI4mm values recorded at a pullback speed of 1.0 mm/sec were not investigated. Therefore, it is not clear if the difference in the maxLCBI4mm value changes in steps as the pullback speed increases. Fifth, the clinical implications of the results in this study need to be verified in the future.

## Conclusions

In a substantial proportion of patients, the location of maxLCBI4mm values measured at 0.5 mm/sec and 2.0 mm/sec was mismatched. In the image sets with matched maxLCBI4mm location, the maxLCBI4mm value at 2.0 mm/sec was significantly greater compared with that at 0.5 mm/sec. These results should be noted when applying the cut-off values of maxLCBI4mm recorded at 2.0 mm/sec.

## Data Availability

All data supporting the findings of this study are available from the corresponding author upon reasonable request.
